# Cryptic diversity of the bent-wing bat, *Miniopterus schreibersii *(Chiroptera: Vespertilionidae), in Asia Minor

**DOI:** 10.1186/1471-2148-10-121

**Published:** 2010-04-30

**Authors:** Andrzej Furman, Tomasz Postawa, Tunç Öztunç, Emrah Çoraman

**Affiliations:** 1 Institute of Environmental Sciences, Boğaziçi University, 34342 Istanbul, Turkey; 2 Institute of Systematics and Evolution of Animals, Polish Academy of Science, Sławkowska 17, 31-016 Kraków, Poland

## Abstract

**Background:**

Two or more species are cryptic, if they are morphologically similar, biologically distinct, and misclassified as a single species. Cryptic species complexes were recently discovered within many bat species and we suspect that the bent-wing bat, *Miniopterus schreibersii*, found in Europe, northern Africa, and Asia Minor, could also form such a complex. Populations of *M. schreibersii *decline in most of the European countries and the species is currently listed as Near Threatened in the IUCN Red List. Finding that *M. schreibersii *is not a single species, but a species complex, would have a considerable impact on its conservation strategies, as the abundance of each component taxon would be much smaller than the one estimated for the nominal species.

**Results:**

*Miniopterus schreibersii *in Asia Minor consists of two genetically diverged lineages, which are reciprocally monophyletic on three mitochondrial DNA markers, have a diagnostic set of multilocus allele frequencies, and show a marked difference in their population structures. The lineages differ slightly in their size, wing shape, and echolocation call parameters. Although these differences are sufficient to discriminate between the lineages, they are not fully diagnostic in reference to individuals. We suggest that the lineages endured the major Northern Hemisphere glaciations in different glacial refugia and colonized Asia Minor after the last glacial maximum. The lineages are allopatric, which is neither delineated by the presence of geographical barriers nor associated with the specific climatic conditions, and which we link to competitive exclusion.

**Conclusions:**

The distinctions between the lineages comply with most of the criteria required for species delineation imposed by various species concepts. Accordingly, we conclude that *M. schreibersii *in Asia Minor is represented by two cryptic species. Our results imply that the distributional range of the nominal species is almost exclusively limited to Europe and the coastal zones of Asia Minor. As populations of *M. schreibersii *seem to be much smaller than currently assumed, conservation strategies regarding this taxon need to be revised. The exact distributional range and the vulnerability of the suggested sister species to *M. schreibersii *is yet to be assessed.

## Background

Two or more species are cryptic, if they are morphologically similar, biologically distinct, and misclassified as a single nominal species [1]. Identification of cryptic species is particularly important in terms of conservation efforts and biodiversity assessments. Species that are apparently in no need of protection may in fact consist of several cryptic and vulnerable component taxa. Similarly, the species richness of some habitats can be considerably underestimated, lowering their conservation status, because of unrecognized cryptic diversity. Identification of cryptic species often starts with a discovery of diverged matrilineal lineages and the typical sequence differences between intra- and interspecific lineages have been quantified for the cytochrome-b gene (*Cytb*) [2]. To delineate species, however, the evidence from mitochondrial DNA has to be accompanied by the supportive data from nuclear markers, detailed phenotypic examination, and geographical distribution analyses.

Speciation without morphological divergence is usually linked to selection promoting morphological invariance or to the use of nonvisual mating signals [1]. Thus it is not surprising that cryptic diversity is particularly common among bats, which communicate their reproductive signals through acoustic calls [3]. Only over the recent few years DNA sequence analyses revealed the presence of 14 previously unrecognized bat species in the western Palaearctic [4,5].

In this study we investigate the bent-wing bat, *Miniopterus schreibersii *(Kuhl, 1817). Up to recently, *M. schreibersii *was considered to be a cosmopolitan species with a near-global distribution [6]. A number of molecular studies, however, proved that *M. schreibersii *was a species complex [7-9] and its presently recognized distributional range is limited to Europe, northern Africa, and Asia Minor [10]. With stable populations in the Balkans and Turkey, and declining in most of the European countries, *M. schreibersii *is listed as Near Threatened in the IUCN Red List [10]. Yet, even within its constrained distributional range, *M. schreibersii *might not be a single nominal species and, in consequence, its populations might be much smaller than currently thought.

In Asia Minor, *M. schreibersii *consists of two morphologically similar but genetically diverged matrilineal lineages, which might be indicative of a cryptic species complex. The lineages are reciprocally monophyletic and differ by ca. 9% on the mitochondrial control region [11,12], ca. 6% on the NADH dehydrogenase subunit 2 (ND2) and ca. 4% on *Cytb *[9]. Calculations based on the ND2 and *Cytb *data estimate that the lineages split ca. 2.0 to 0.5 Myr BP, implying separation in disjunct Pleistocene glacial refugia. The lineages are allopatric and separated along a zone passing through Central Anatolia [12].

To examine the hypothesis that *M. schreibersii *in Asia Minor forms a cryptic species complex, we employ a multidisciplinary approach. We use molecular markers to analyze the distribution of the lineages, their population structure and reproductive isolation. We examine morphological and echolocation data to measure ecological divergence of the lineages. Because allopatric populations are *per se *reproductively isolated, we focus on the putative contact zone between the lineages in Central Anatolia, where the probability of finding mixed colonies or interbreeding individuals is the highest. The sampled roosts are located within different climatic regimes and separated from each other by distances, which *M. schreibersii *is able to cover in its migratory movements [13,14]. The location of the roosts allows us to examine the suggestion that the allopatric distribution of the lineages is caused by different climatic preferences [11].

## Methods

### Sampling

**Figure 1:**
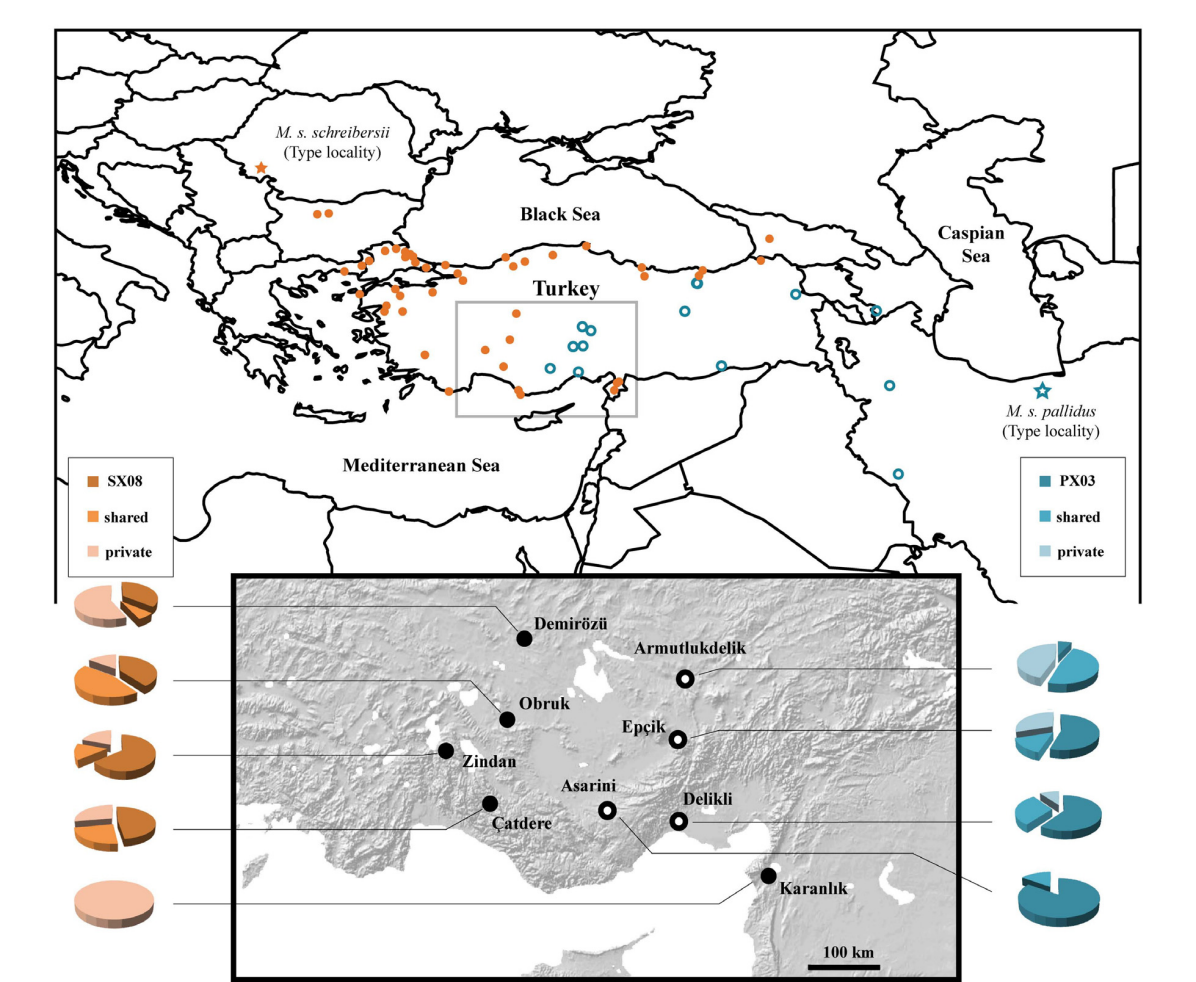
**Genetically identified colonies of *M. s. schreibersii *(filled circles) and *M. s. pallidus *(empty circles) in Thrace and Asia Minor**. The underground sites sampled in this study are marked in the insert. Pie charts show distribution of the most common, shared with other colonies, and private haplotypes.

Samples were collected during summer in nine underground sites. The sites were located in the area of about 500 km by 400 km in the south of Turkey, which covered parts of the Central Anatolian Plateau and the Mediterranean Sea coast (Figure 1). In each site, ca. 20 bats were caught by a hand net. Bats were sexed, measured, and their right wing photographed. Some individuals had their echolocation calls recorded. Tissue samples were collected from the wing membrane by 3 mm biopsy-punchers as outlined by Worthington Wilmer & Barratt [15] and stored in 80% ethyl alcohol. After sampling, bats were immediately released. Our research was approved by the Boğaziçi University Ethics Committee on Animal Research (BÜHADYEK).

### Mitochondrial and Microsatellite Markers

Genomic DNA was extracted from biopsy punches following the Roche High Pure PCR Template Preparation Kit protocol. The mitochondrial control region was amplified with two primers, L15408 and E following the procedures described in Irwin *et al*. [16] and Wilkinson & Chapman [17]. PCR products were purified and sequenced with primer E in Macrogen Inc. Korea. The resulting 152 sequences were edited with Sequencher v. 4.9 (Genecodes Corp., Ann Arbor, MI, USA); the percent of quality bases for each of the sequences varied from 90.2 to 99.8% and the average quality score was 97.2%. The sequences were aligned using Clustal × v. 1.8 [18] and inspected visually. The sequences representing unique haplotypes have been deposited to GenBank with the accession numbers [HM044071-HM044103]. Our data were analyzed together with 39 sequences reported previously [11,12].

Microsatellite loci were amplified with ten pairs of primers, *Mschreib2*, *Mschreib4*, *CH37*, *CH85*, *CH87*, *CHA14*, *CHB12*, *CHD2*, *CHD3*, and *NCAM *following the procedures described in Miller-Butterworth *et al*. [19], Han *et al*. [20], and Moore *et al*. [21]. Four loci, *CH85*, *CHA14*, *CHD3*, and *NCAM*, were monomorphic and were excluded from further analyses. Microsatellite loci were scored in Macrogen Inc. Korea. To detect the presence of possible errors in our data we used Micro-Checker 2.2 [22]. No null alleles, large allelic dropout, or other errors were identified.

### Lineage Identification

We refer to the matrilineal lineages of *M. schreibersii *as *M. s. schreibersii *(type locality: Kolumbacs cave, Romania; Kuhl, 1817) and *M. s. pallidus *(type locality: South coast of Caspian Sea, Iran; Thomas, 1907). Our delineation of the lineages is not based on a difference in dorsal coloration, which traditionally was used to distinguish these subspecies but proved to be inconsistent with their genetic identity [12]. Instead, we refer to the similarity of the mitochondrial control region sequences of *M. schreibersii *from Bulgaria (located near the type locality of *M. s. schreibersii*) to one of the lineages and the sequences of *M. schreibersii *from Iran and Nagorno-Karabakh (located near the type locality of *M. s. pallidus*) to the second one. The lineages can be easily identified by the ten-base fragment, which starts in the beginning of mitochondrial control region (at the seventh position after tRNA-Pro). The fragment 'C-TGTCAAGT' is typical for *M. s. schreibersii *and the fragment 'TATGCTGGAC' is typical for *M. s. pallidus*. These fragments have been invariant in all sequences of the mitochondrial control region examined by us up to now.

### Mitochondrial and Nuclear Data Analyses

DNA polymorphism of the mitochondrial control region was assessed by nucleotide diversity and haplotype diversity. Genetic divergence was measured by the average number of pairwise nucleotide differences and the average number of nucleotide substitutions per site with the Jukes and Cantor correction (JC) [23,24]. The relevant statistics were computed with DnaSP v. 5.00.07 [25] and Arlequin 3.11 [26]. We used the JC estimate, because the number of nucleotide substitutions per site was less than 0.1, in which case all correction methods calculate about the same distances as JC for closely related sequences [27]. Genetic diversity in the microsatellite data was evaluated by the mean alleles number and the proportion of heterozygotes; both calculated with GeneClass2 [28]. GeneClass2 was also used to implement an assignment test with Bayesian computational criteria [29].

Population genetic structure was investigated by analysis of molecular variance (AMOVA) with *F *(microsatellites) or Φ statistics (mtDNA control region); significance of the fixation indices was assessed with 50 000 permutations with Arlequin v. 3.11. Correlations between genetic divergences and geographic distances were analyzed by plotting pairwise Rousset's distance (Φ_ST_/[1- Φ_ST_] or *F*_ST_/[1- *F*_ST_]) against the logarithm of geographical distance [30]. Statistical significance of correlations was assessed by a Mantel test with 10,000 randomizations as implemented in IBDWS v. 3.15 [31]. Genealogical relationships among haplotypes based on the mitochondrial data were estimated by a statistical parsimony network using 95% connection limit [32] with TCS v. 1.21 [33].

Demographic history was inferred from a mismatch distribution analysis [34] and raggedness statistics [35]. The models' goodness of fit and the population expansion time parameters (*τ*) were assessed with 10,000 bootstrap replicates using Arlequin v. 3.11. Approximate time to population expansion events was estimated with the assumption of a mutation rate of 20% per Myr [36] and a generation time of five years [14]. Although the assumed mutation rate was calibrated for D-loop in *Nyctalus noctula*, it was also used to estimate population expansion times for *M. schreibersii *[11,12,14]. Additionally, the approximation of 20% per Myr represents the average mutation rate estimated for other mammals, which varies from 10% per Myr for house mouse [37], through 12-17% per Myr for humans [38], to 30% per Myr for steppe bison [39].

Phylogenetic trees were constructed with Neighbour Joining method (NJ) [40]. We used the NJ method as it can easily accommodate short internal branches and is recommended for constructing phylogenetic trees of closely related species [41]. For the mitochondrial data, the tree was generated with PAUP* v. 4.0b10 [42]. In the mitochondrial control region analysis we included four sequences from Bulgaria [GenBank: EU332359, EU332360, EU332369, and EU332378], two sequences from Iran [GenBank: FJ028638 and FJ028640], and two sequences from Nagorno-Karabakh [GenBank: FJ028634 and FJ028635], which represent samples collected near the type localities of *M. s. schreibersii *and *M. s. pallidus *[11,12]. For the microsatellite data, the tree was constructed with Poptree (N. Takezaki, Max-Planck Institut für Biologie, Tuebingen, Germany) using the modified Cavalli-Sforza distance [43]. The nodal support was assessed from 10,000 nonparametric bootstrap replicates.

### Morphometric and Echolocation Data

Morphometric measurements consisted of body mass (precision: ± 0.5 g) and wing measurements (precision ± 0.1 mm). The wing measurements included forearm length (FA) measured on captured bats and phalanges length of the third, fourth, and fifth digit measured on wing photographs. Juvenile bats were excluded from analyses. We used wing measurements to compute tip index, aspect ratio index, and area index as described in Findley *et al*. [44]. Morphological wing data were analyzed with a t-test and a stepwise discriminant function analysis.

Echolocation calls were recorded in a flight tent (5 m × 1.5 m × 1.5 m) and/or from hand-released bats with a time expansion bat detector (D240x, Pettersson Elektronik AB, Uppsala, Sweden) using a sampling frequency of 44.1 kHz with 16 bits/sample. Only the search-phase calls were recorded. The calls, after frequency transformation, were stored on a digital sound recorder (Edirol R-09, Roland Corporation). The recordings were analyzed with BatSound v. 3.31 (Pettersson Elektronik AB, Uppsala, Sweden). The analyzed echolocation call parameters included starting frequency, terminal frequency, peak frequency, band width (a difference between starting and terminal frequencies), and call duration. For each bat, the echolocation call parameters were averaged from the five best quality recordings. The echolocation parameters were analyzed by a t-test and a stepwise discriminant function analysis.

## Results

### Distribution

*Miniopterus s. schreibersii *and *M. s. pallidus *were strictly allopatric within their putative contact zone. The colonies of both lineages were found on the Central Anatolian Plateau with the semiarid steppe climate and on the Mediterranean Sea coast with the wet Mediterranean climate. Similarly, there was no distinction in the altitude of the roost occupied by *M. s. schreibersii *or *M. s. pallidus*, which in both cases were located on elevations varying from ca. 200 m to 1600 m Above Sea Level.

### Mitochondrial and Microsatellite Data

The 191 mitochondrial control region sequences (110 *M. s. schreibersii *and 81 *M. s. pallidus*), were 418 or 419 bp long. The sequences of *M. s. pallidus *were longer because of a base insertion at the ninth position after Pro-tRNA gene. The sequences yielded 43 unique haplotypes: 27 in *M. s. schreibersii *and 16 in *M. s. pallidus *(Additional file 1). The average haplotype diversity within *M. s. schreibersii *was 0.84 ± 0.03 and 0.71 ± 0.05 within *M. s. pallidus*; the average nucleotide diversities (JC) were 0.0067 ± 0.0004 and 0.0036 ± 0.0004, respectively. There were 19 fixed differences between *M. s. schreibersii *and *M. s. pallidus*, 25 substitutions were polymorphic in *M. s. schreibersii*, but monomorphic in *M. s. pallidus*, 9 substitutions were polymorphic in *M. s. pallidus*, but monomorphic in *M. s. schreibersii*, and 4 substitutions were shared. The average number of nucleotide differences was 29.77 and the average number of nucleotide substitutions per site (JC) between *M. s. schreibersii *and *M. s. pallidus *was 0.075 ± 0.005.

The six polymorphic microsatellite loci scored for 174 individuals (95 *M. s. schreibersii *and 79 *M. s. pallidus*) yielded 57 distinct alleles. The mean allele richness was 6.33 ± 3.67 in *M. s. schreibersii *and 8.00 ± 3.23 in *M. s. pallidus*; the mean heterozygosities were 0.42 ± 0.31 and 0.63 ± 0.23, respectively. When only a single locus was analyzed, an assignment test with the threshold of scores set to 0.05 correctly identified from 80% (*Mschreib4*) to 98% (*CH37*) of individuals as *M. s. schreibersii *or *M. s. pallidus*. With all six microsatellite loci, the test correctly identified 100% of individuals (quality index = 99.92%), indicating that each lineage had a diagnostic set of multilocus allele frequencies.

**Figure 2:**
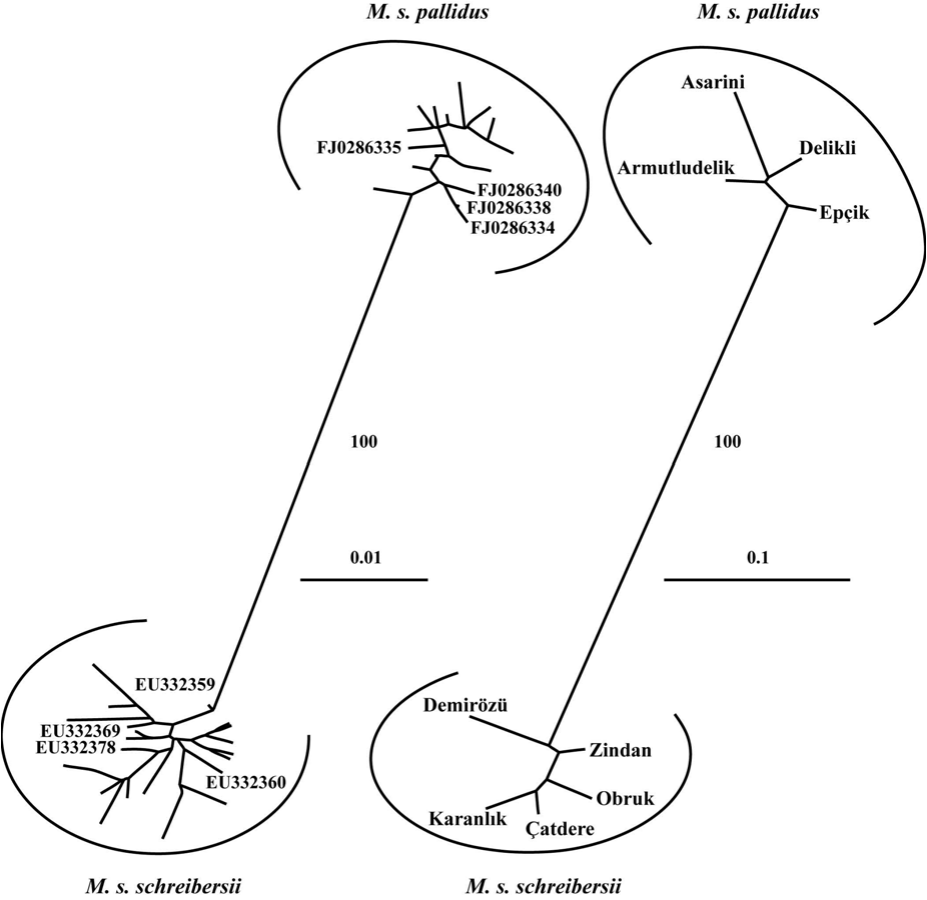
**Phylogenetic NJ trees constructed from the mtDNA (left) and microsatellite (right) data**. Accession mark GenBank sequences of *M. s. schreibersii *from Bulgaria (EU332359, EU332360, EU332369, and EU332378) and GenBank sequences of *M. s. pallidus *from Iran (FJ028638 and FJ028640) and Nagorno-Karabakh (FJ028634 and FJ028635). The nodal support is shown only for the main lineages.

The unrooted NJ tree constructed from microsatellite data clearly separated colonies of *M. s. schreibersii *and *M. s. pallidus *with 100% bootstrap support (Figure 2). Similarly, the tree based on the mitochondrial data also recovered two distinct lineages of *M. s. schreibersii *and *M. s. pallidus *with 100% bootstrap support (Figure 2). The *M. s. schreibersii *clade included the sequences from Bulgaria and the *M. s. pallidus *clade included the sequences from Iran and Nagorno-Karabakh.

### Population Genetic Structure

The average haplotype diversity within the colonies of *M. s. schreibersii *varied from 0.58 to 0.77 and the average nucleotide diversity varied from 0.0035 to 0.0069 (Table 1). The colonies showed only a moderate genetic differentiation with 74% of the molecular variance explained by within colonies diversity (Φ_ST _= 0.257, *p *< 0.001). Pairwise fixation indices were larger between Karanlık and other colonies (0.352 to 0.478) than between the remaining pairs (≤ 0.226) (Table 2). Rousset's distances calculated for pairs of the *M. s. schreibersii *colonies strongly correlated with the logarithm of geographic distances between them (*r *= 0.91; *p *< 0.001). Within the colonies of *M. s. pallidus*, the average haplotype diversity varied more and the average nucleotide diversity was lower (0.28 to 0.84 and 0.0014 to 0.0041, respectively). The colonies of *M. s. pallidus *also revealed a much weaker population structure; 92% of the molecular variance was assigned to within colonies diversity (Φ_ST _= 0.081, *p *= 0.003). All, but one, fixation indices between the colonies of *M. s. pallidus *were smaller than 0.1. The only exception was the Armutludelik-Asarini pair; the two farthest colonies (Φ_ST _= 0.257). Although there was a considerable correlation between Rousset's distances and the logarithm of geographic separation between pairs of the *M. s. pallidus *colonies, the relation was not significant (*r *= 0.74; *p *= 0.084).

**Table 1:** Genetic diversity within the colonies of *M. s. schreibersii *and *M. s. pallidus *based on 418/419 bp of the mtDNA control region sequences and six microsatellite loci.

		mtDNA				microsatellites		
	**Site**	**N**	**h**	***H*_d_**	***π*(JC)**	**N**	**A**	***H*_E_**

*M. s. schreibersii*	Çatdere	25	10	0.77	0.0069	11	3.333	0.379
	Demirözü	14	4	0.66	0.0059	14	4.333	0.495
	Karanlık	31	9	0.73	0.0035	30	4.000	0.426
	Obruk	20	6	0.71	0.0060	20	4.333	0.408
	Zindan	20	6	0.58	0.0047	20	4.500	0.375

*M. s. pallidus*	Armutludelik	21	8	0.84	0.0041	20	5.500	0.581
	Asarini	20	4	0.28	0.0014	20	6.333	0.611
	Delikli	20	6	0.63	0.0039	20	5.833	0.644
	Epçik	20	10	0.71	0.0040	19	6.500	0.671

N = number of individuals sampled, h = number of observed haplotypes, *H*_d _= haplotype diversity, *π*(JC) = nucleotide diversity with the Jukes and Cantor correction, A = mean alleles number, *H*_E _= proportion of heterozygotes.

**Table 2:** Pairwise fixation indices between the colonies of *M. s. schreibersii *and *M. s. pallidus *derived from the mtDNA data (Φ_ST_; below diagonal) and from the microsatellite data (*F*_ST_; above diagonal).

*M. s. schreibersii*	Çatdere	Demirözü	Karanlık	Obruk	Zindan
Çatdere	-	0.03330^(*)^	0.01621	0.00207	-0.01506
Demirözü	0.15715^(*)^	-	0.04450^(*)^	0.02673^(*)^	0.02648^(*)^
Karanlık	0.36601^(*)^	0.35221^(*)^	-	0.02196^(*)^	0.05238^(*)^
Obruk	0.07481	0.09962	0.35343^(*)^	-	0.01712
Zindan	-0.00403	0.22607^(*)^	0.47821^(*)^	0.12523^(*)^	-

*M. s. pallidus*	Armutludelik	Asarini	Delikli	Epçik	

Armutludelik	-	0.01441	0.00059	0.00590	
Asarini	0.25711^(*)^	-	-0.00127	- 0.00033	
Delikli	0.07295^(*)^	0.03930	-	-0.00452	
Epçik	0.08666^(*)^	0.01081	-0.01831	-	

Stars indicate values significant at the 0.05 level.

The mean number of microsatellite alleles within the colonies of *M. s. schreibersii *varied from 3.33 to 4.50 and the proportion of heterozygotes ranged from 0.38 to 0.50 (Table 1). There was only a slight genetic differentiation among the colonies of *M. s. schreibersii *and 97% of the molecular variation was explained by within colony diversity (*F*_ST _= 0.027, *p *< 0.001). Within the colonies of *M. s. pallidus *both the mean number of alleles, and the proportion of heterozygotes were higher than in *M. s. schreibersii*: 5.50 to 6.50 and 0.58 to 0.67, respectively. The colonies of *M. s. pallidus *did not show any indications of gene flow restrictions and almost 100% of molecular variance was explained by within colony diversity (*F*_ST _= 0.002, *p *= 0.307). Pairwise differences (*F*_ST_) between the colonies of *M. s. schreibersii *were very small, signifying only weak population structure (Table 2). Rousset's distances calculated for pairs of the colonies of *M. s. schreibersii *strongly correlated with the logarithm of geographic distances between them (*r *= 0.69; *p *= 0.020). The colonies of *M. s. pallidus *showed no between-colony differentiations as indicated by pairwise fixation indices being about one order of magnitude smaller than those observed in *M. s. schreibersii*. Similarly, there was no correlation between Rousset's distances and the logarithm of geographic distances in *M. s. pallidus *(*r *= -0.06; *p *= 0.627).

### Statistical Parsimony Network

**Figure 3:**
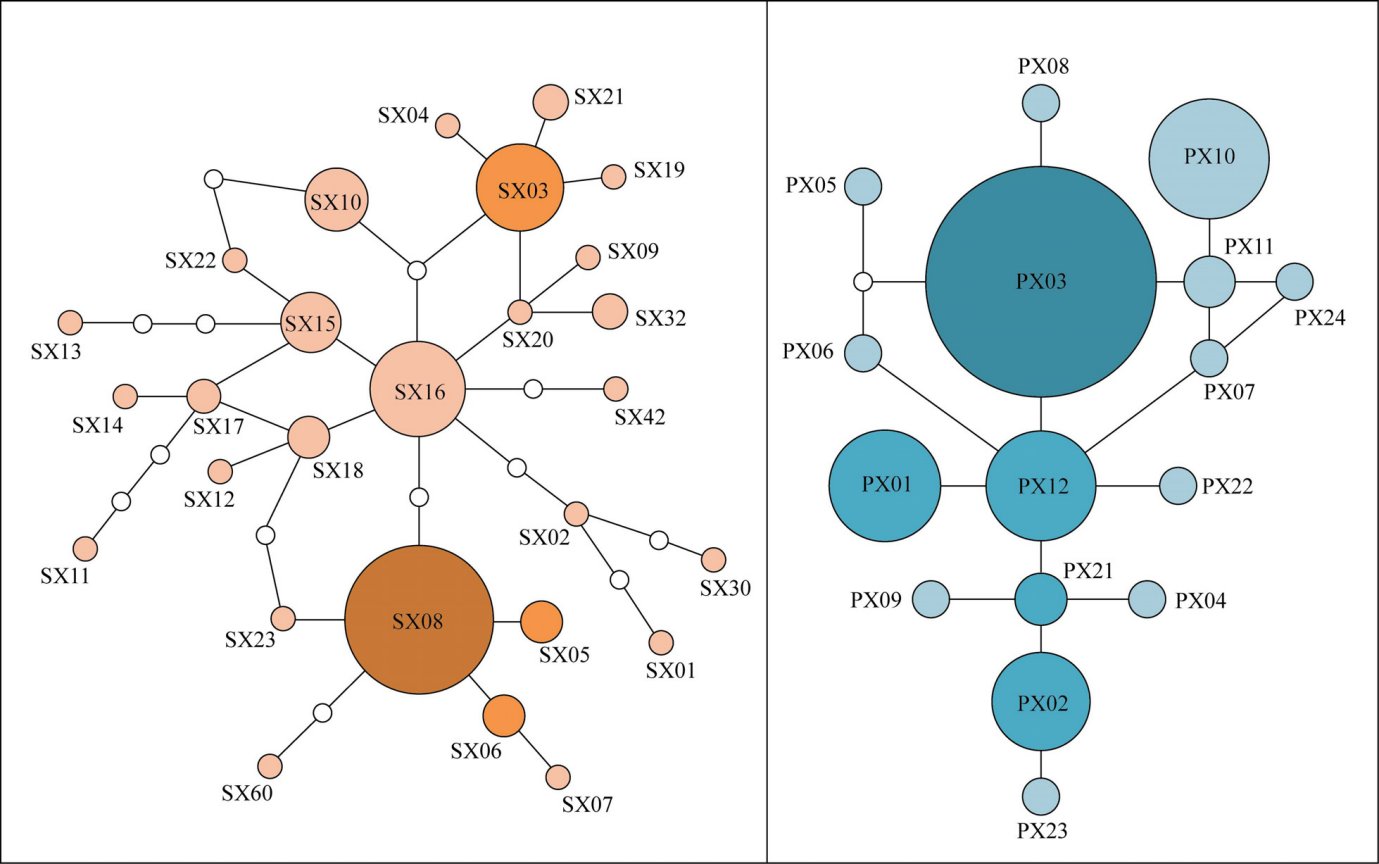
**Statistical parsimony network: genealogical relationships among haplotypes of *M. s. schreibersii *(left) and *M. s. pallidus *(right)**. The most common, shared with other colonies, and private haplotypes are shaded in the same way as in Figure 1. Small, empty circles indicate intermediate haplotypes that were not present in the sample. Lines in the network represent single mutational changes. Sizes are proportional to the number of individuals.

A statistical parsimony network of *M. s. schreibersii *was built around the most common haplotype SX08 (38 individuals; 35%; absent in Karanlık) (Figure 3). Because of its central position in the network, high number of connections, and a spacious distribution, SX08 was accepted to be the most plausible candidate for the ancestral haplotype of *M. s. schreibersii*. Although SX03 and SX16 also had many connections to other haplotypes, SX03 had a marginal position in the network and SX16 was spatially constrained to Karanlık. Additionally, SX16 was linked to other colonies almost entirely through 'absent-haplotype' connections. A statistical parsimony network of *M. s. pallidus *was constructed around the central haplotype PX12 (9 individuals; 10%; absent in Delikli). Although this haplotype was neither the most common nor the most widespread, its central position and connections to six other haplotypes made it a likely candidate to be the ancestral haplotype within the *M. s. pallidus *colonies. There was a pronounced southward decrease in haplotype diversity within the colonies of *M. s. pallidus*: the most common hyplotype, PX03, was present in 7% of individuals in Armutludelik, 55% in Epçik, 60% in Delikli, and 85% in Asarini (Figure 1). Correspondingly, frequencies of private haplotypes were 45%, 30%, 10%, and 0%, respectively.

### Mismatch Distribution

The mismatch distribution analysis suggested population expansion models for demographic histories of *M. s. schreibersii *and *M. s. pallidus*. Harpending's raggedness indices were *r*_*M.s.s*. _= 0.065 and *r*_*M.s.p*. _= 0.044. In the demographic expansion models, the probabilities of observing distributions with higher raggedness under a null hypothesis of population expansion were *p*_*M.s.s*. _= 0.11 and *p*_*M.s.p*. _= 0.73. The sums of squared differences between the observed and expected distributions were no significant (*p*_*M.s.s*. _= 0.15; *p*_*M.s.p*. _= 0.45). The estimated time of the demographic expansion was 21.5 kyr BP (95% C. I.: 36.5 to 6.6 kyr BP) for *M. s. schreibersii *(*τ *= 3.6) and 13.7 kyr BP (95% C. I.: 29.2 to 0.4 kyr BP) for *M. s. pallidus *(*τ *= 2.3).

### Morphometrics

The body mass was taken in 133 individuals (73 *M. s. schreibersii *and 60 *M. s. pallidus*). *Miniopterus s. schreibersii *was a lighter bat than *M. s. pallidus *(13.0 ± 1.1 g and 14.7 ± 1.2 g, respectively); the difference being significant at the 0.001 level. There were not any significant differences in weight between sexes within each group.

The wing measurements were analyzed for 150 individuals (78 *M. s. schreibersii *and 72 *M. s. pallidus*). The average forearm length of *M. s. schreibersii *(46 females and 32 males) was 45.7 ± 0.6 mm and that of *M. s. pallidus *(22 females and 50 males) was 46.8 ± 0.7 mm, the difference being significant at the 0.001 level. The observed difference in forearm length was almost exactly the same as the one based on much wider distributional sampling [12]. There was a slight sexual dimorphism within both lineages; females tended to have longer forearms than males, but the differences were not statistically significant. Forearm lengths of *M. s. schreibersii *and *M. s. pallidus *were normally distributed. Except for the 1^st ^phalanx of the 3^rd ^digit (3c), the average lengths of phalanges (Table 3) were consistently longer in *M. s. pallidus *(*p *< 0.001). The 3c phalanx length was approximately the same in both groups (*p *= 0.323). All wing indices differed significantly at the 0.001 level (tip index: TI_*M.s.s*. _= 1.99 ± 0.03 and TI_*M.s.p*. _= 1.96 ± 0.03; aspect ratio index: AR_*M.s.s*. _= 2.53 ± 0.06 and AR_*M.s.p*. _= 2.45 ± 0.04; area index AI_*M.s.s*. _= 14756 ± 554 and AI_*M.s.p*. _= 15567 ± 481). Stepwise discriminant analysis, using the phalanges and forearm measurements, found a canonical function that included three variables: forearm length, length of the 2^nd ^phalanx of the 5^th ^digit, and length of the metacarpal of the 4^th ^digit; the discriminant function correctly assigned the group membership in 85% of the individuals.

**Table 3:** Length of the phalanges of the 3^rd^, 4^th^, and 5^th ^digit (a: the metacarpal; b: the 2^nd ^phalanx; c: the 1^st ^phalanx) ([mm]; mean ± SD).

	3a	3b	3c	4a	4b	4c	5a	5b	5c
*M. s. s*.	42.4 ± 0.7	10.8 ± 0.3	37.4 ± 1.0	41.2 ± 0.7	8.80 ± 0.3	16.9 ± 0.6	37.5 ± 0.9	8.4 ± 0.9	8.2 ± 0.8

*M. s. p*.	43.0 ± 0.7	11.2 ± 0.4	37.2 ± 1.0	42.0 ± 0.7	9.0 ± 0.3	17.7 ± 0.7	38.2 ± 0.7	9.3 ± 0.5	8.9 ± 0.6

### Echolocation

The echolocation call parameters were analyzed in 33 individuals (13 *M. s. schreibersii *and 20 *M. s. pallidus*). The starting frequency, the peak frequency, and the band width differed significantly between the lineages at the 0.001 level (Table 4). The terminal frequency and the call duration were similar in both groups (*p*_duration _= 0.294 and *p*_terminal _= 0.270). A stepwise discriminant analysis found a canonical function that included two variables, the starting frequency and the call duration; the discriminant function assigned 88% of the individuals to the correct groups.

**Table 4:** Echolocation calls parameters: SF = starting frequency, TF = terminal frequency, PF = starting frequency, BW = band width, CD = call duration.

		SF [kHz]	TF [kHz]	PF [kHz]	BW [kHz]	CD [ms]
*M. s. schreibersii*	Mean ± S D	111.1 ± 4.4	48.6 ± 1.5	58.8 ± 1.7	62.7 ± 4.9	3.4 ± 0.5
	Range	104.8 - 118.3	46.3 - 52.0	56.4 - 61.7	56.5 - 71.8	2.8 - 4.6

*M. s. pallidus*	Mean ± S D	95.9 ± 8.2	48.1 ± 0.9	56.1 ± 1.5	47.8 ± 8.5	3.6 ± 0.8
	Range	72.7 - 107.2	46.4 - 49.5	53.1 - 58.5	25.3 - 61.0	2.3 - 5.6

## Discussion

Cryptic species complexes, in which the component taxa have not diverged morphologically too much, are very difficult to identify and their discovery is frequently a matter of chance. In a recent study investigating the contribution of the 28 Iberian bat species to the cryptic diversity in Europe, almost 20% of the Iberian bats showed considerable mitochondrial discontinuities either within the Iberian or between Iberian and other European samples [4]. One of the very few species, which in that study proved to be genetically very homogenous, was *M. schreibersii*. In Asia Minor, however, *M. schreibersii *consists of two genetic lineages, which differ considerably on three mitochondrial markers (*Cytb*, ND2, and the control region) and which have the diagnostic set of multilocus allele frequencies [9,12, this study]. The mitochondrial differentiation between the *M. schreibersii *lineages is particularly striking in the light of the similarity observed within each of them; there is only a shallow differentiation in the control region and almost no differentiation in *Cytb *between colonies of *M. s. schreibersii *sampled in Anatolia, Iberia, or Maghreb [4,9,45]. A similar homogeneity is observed within *M. s. pallidus *[9].

### Population Genetic Structure

The extant colonies of *M. s. schreibersii *and *M. s. pallidus *in Anatolia show visible differences in their population genetic structure. The colonies of *M. s. schreibersii *reveal the moderate differentiation in the control region sequences accompanied by the very strong correlation between the genetic and geographic distances. At the microsatellite level, the differentiation is weak but the association between the genetic and geographic distances is still strong. The results are in concordance with the ones reported for *M. s. schreibersii *from Portugal, which were linked to female philopatry and a mainly male-mediated gene flow [14]. On the other hand, the colonies of *M. s. pallidus *show only a weak differentiation in the control region sequences and no significant correlation between the genetic differentiation and geographic distance. The latter outcome, however, should be taken with a caution as the small number of colonies could profoundly affect the significance of the correlation. At the microsatellite level, the colonies of *M. s. pallidus *do not reveal any indications of population structuring. Both the differentiation between the colonies and the association between the genetic and geographic distances are virtually zero, suggesting no constrains imposed on gene flow.

The colonies of *M. s. schreibersii *in Karanlık and *M. s. pallidus *in Asarini and Delikli deserve a special attention (Figure 1). The former one is distanced from the other colonies of *M. s. schreibersii *by about 400-500 km and interposed by the colonies in Asarini and Delikli, which mark the south-most border of the Anatolian distribution of *M. s. pallidus*. The colony in Karanlık accommodates only private haplotypes, a signature of an isolated population. Although the intermediate haplotypes connecting Karanlık to other colonies are mostly missing, Karanlık's haplotypes occupy the central position in the statistical parsimony network, suggesting a relatively recent demographic history shared with the other *M. s. schreibersii *colonies. On the other hand, the colony in Asarini accommodates only 10% of private haplotypes and Delikli accommodates none. The pattern of decrease in frequency of private haplotypes from the Central Anatolian Plateau towards the Mediterranean, complemented by the increase in frequency of one haplotype, PX03, is observed in all of the *M. s. pallidus *colonies. This pattern may indicate the recent southwards expansion from Central Anatolia. Such expansion would include many founder/bottleneck episodes and the successful vanguards would dominate the new population genome, a chain of events inevitably leading to a loss of haplotype diversity [46,47].

### Morphology

Morphologically, *M. s. schreibersii *is a smaller bat than *M. s. pallidus *(shorter forearm length and lower wing area index) and has narrower and more elongated wings (higher tip and aspect ratio indices). There are also significant differences in echolocation call parameters between the lineages. Although, in principle, higher aspect ratio and tip indices are associated with swifter flight [44] and variations in echolocation calls are related to utilization of diverse foraging habitats [48], the differences found between *M. s. schreibersii *and *M. s. pallidus *are relatively small and the ranges are overlapping. Indeed, even though wing morphology and echolocation call parameters are sufficient to discriminate between the lineages, they are not fully diagnostic in reference to a single individual. In consequence, the observed dissimilarities can probably only minutely affect the foraging performance of bats, resulting in a considerable overlap in their ecological niches and leaving them prone to an intensive inter-lineage competition. A similar situation is found in European *Plecotus *species: echolocation signals of *P. macrobullaris *are more similar to allopatrically occurring *P. austriacus *than to co-occurring *P. auritus*, although *P. macrobullaris *is genetically closer related to *P. auritus *[49,50]. In fact, morphological similarity is typical for all species historically comprising the *M. schreibersii *complex [7] and may indicate intrinsic deficiency in phenotypic plasticity within these taxa or selection promoting morphological stasis.

### Historical Scenario

According to the estimates based on the *Cytb *and ND2 data, *M. s. schreibersii *and *M. s. pallidus *diverged after the onset of the major Northern Hemisphere glaciations in the Lower or Middle Pleistocene [9]. The lineages probably remained isolated until the end of the last glacial maximum. The best estimates imply that *M. s. schreibersii *survived the major glaciations in a single glacial refugium in the north-western Anatolia/Balkans and rapidly colonized Europe ca. 15 kyr BP [51]. Asia Minor was probably colonized earlier by *M. s. schreibersii *and a few colonies might have survived the last glacial maximum in one of the south Anatolian refugia, located along the Mediterranean Sea coast. The latter hypothesis is supported by the earlier expansion time estimated here for the south Anatolian population (ca. 21 kyr BP) than for the European expansion. Considerably less can be conjectured about the history *M. s. pallidus*; it probably survived the major glaciations in refugia located somewhere on the southern coast of the Caspian Sea, the eastern part of the Caucasian refugium. It is unlikely that *M. s. pallidus *endured the glacial periods in Colchis, a western part of the Caucasian refugium, as this region is presently occupied by *M. s. schreibersii *and it is dubious that the former replaced the latter. *Miniopterus s. pallidus *possibly expanded to Anatolia after the climatic change that followed the end of the last glacial maximum, ca. 13 kyr BP. The late colonization of the Central Anatolian Plateau may be a result of the high altitude of the region (and more severe climatic conditions) and the scarcity of suitable underground habitats. The lineages came to contact in the Central Anatolia. Probably, when they met, *M. s. schreibersii *had already established colonies on the Central Anatolian Plateau and along the Mediterranean Sea coast.

### Distribution

Spatial separation of *M. s. schreibersii *and *M. s. pallidus *is neither delineated by the presence of geographical barriers nor associated with the specific climatic conditions. Although most of the known colonies of *M. s. schreibersii *are found in coastal, low altitude locations with the wet Mediterranean or Black Sea climate [11,12], the colonies in Obruk and Demirözü are located in the Central Anatolian Plateau with the semiarid steppe climate on the altitudes of ca. 1600 m and 900 m, respectively. Conversely, even though *M. s. pallidus *mainly occupies semiarid regions of higher altitude, the colonies in Asarini and Delikli are located near, or on, the Mediterranean Sea coast exhibiting the wet Mediterranean climate, on the altitudes of ca. 1600 m and 200 m. Therefore, it is plausible to conclude that both lineages are fully capable of utilizing both inland and coastal habitats, and have similar ecological niches.

We suggest that in its southwards expansion, *M. s. pallidus *possibly replaced some colonies of *M. s. schreibersii*. The replacement rather than a sympatric coexistence of the lineages could be caused by the morphological similarities between the lineages and resulting competitive exclusion [52]. Accordingly, we presume that the extant geographical distributions of *M. s. schreibersii *and *M. s. pallidus *are the result of the historical and ongoing expansion events, and interlineage competition rather than the outcome of climatic preferences as suggested by Bilgin *et al*. [11].

### Taxonomical Implications

In the light of available evidence, *M. s. schreibersii *and *M. s. pallidus *form two separately evolving lineages, making them potentially different species [53]. The lineages are reciprocally monophyletic on the three mitochondrial DNA markers [9,12], have the diagnostic set of multilocus allele frequencies and their common ancestor is apparently extinct, satisfying the main criteria imposed by the phylogenetic species concept [54,55]. The genetic differentiation between lineages in the mitochondrial cytochrome-b gene is within the range recognized for sister taxa by the genetic species concept [2]. The lineages are also phenetically distinguishable, in line with the phenetic species concept [56,57]. Furthermore, the lineages seem to be reproductively isolated, as implied by the divergence on nuclear microsatellites in the putative contact zone, despite the lack of any obvious geographic barriers, partly fulfilling requirements of the biological species concept [58,59].

## Conclusions

The distinctions between *M. s. pallidus *and *M. s. schreibersii *seem to be sufficient to recognize *M. schreibersii *in Asia Minor as a cryptic species complex and to grant *M. s. pallidus *a full species status, a sister taxon to *M. schreibersii*. Still, we do not know about the mechanism of reproductive isolation between *M. s. schreibersii *and *M. s. pallidus*, which probably includes a difference in mating calls, and we know very little about their ecological divergence. Further and more extensive studies are needed to clarify these points.

The results of this study are particularly important for the conservation of the *M. schreibersii*. The distributional range of the nominal species, *M. s. schreibersii*, is mainly limited to Europe and the coastal zones of Asia Minor; an area much smaller than currently recognized by the IUCN Red List of Threatened Species [10]. The rate of population decline of *M. schreibersii*, including stable populations in the Balkans and Turkey, is estimated to be approaching 30% [10]. However, taking into consideration that many Turkish populations represent *M. s. pallidus*, the decline rate of *M. s. schreibersii *might already be much higher than estimated by the IUCN Red List. Accordingly, a thoughtful revision of all conservation strategies regarding *M. s. schreibersii *might be crucial to preserve this taxon. The presence of *M. s. pallidus *in Asia Minor requires a protection program, which would estimate abundance of its current populations, identify possible threats, and assess its conservation status.

Finally, the case of *M. schreibersii *may be indicative for other bat species, which persisted through the major glaciations in the Anatolian refugia. Here, potential candidates are *Rhinolophus euryale *and *R. ferrumequinum*. Both species consist of considerably diverged matrilineal lineages [60,61] and each of them could be, in principle, a cryptic species complex.

## Authors' contributions

AF designed the study, carried out the field work, performed statistical analyses of molecular data, and drafted the manuscript. TP carried out the field work and analyzed morphological and echolocation data. TÖ carried out the field and molecular laboratory work. EÇ participated in the design of the study, carried out the field work and helped in drafting the manuscript. All authors read and approved the manuscript.

## Supplementary Material

Additional file 1 List of haplotypes analyzed in this study. Click here for file
