# Supplementary material for: Cryptic diversity of the bent-wing bat, Miniopterus schreibersii (Chiroptera: Vespertilionidae), in Asia Minor
Source: BMC Evol Biol. 2010 Apr 30;10:121. doi: 10.1186/1471-2148-10-121 (PMC2873419; doi:10.1186/1471-2148-10-121)
Supplement: Additional file 1 — List of haplotypes analyzed in this study. [file 1471-2148-10-121-S1.DOC]

**ADDITIONAL FILE 1 – List of haplotypes analyzed in this study.**

GenBank accession numbers (round brackets), localities, and frequencies (square brackets) of the haplotypes analyzed in this study.

SX01 (HM044071): Çatdere [1]; SX02 (HM044072): Çatdere [1]; SX03 (HM044073): Çatdere [2], Demirözü [1], Obruk [8], Zindan [2]; SX04 (HM044074): Çatdere [1]; SX05 (HM044075): Çatdere [2], Zindan [1]; SX06 (HM044076): Çatdere [2], Obruk [1]; SX07 (HM044077): Çatdere [1]; SX08 (HM044078): Çatdere [12], Demirözü [5], Obruk [8], Zindan [13]; SX09 (HM044079): Demirözü [1]; SX10 (HM044080): Demirözü [7]; SX11 (HM044081): Karanlık [1]; SX12 (HM044082): Karanlık [1]; SX13 (FJ028648): Karanlık [1]; SX14 (FJ028610): Karanlık [1]; SX15 (HM044083): Karanlık [6]; SX16 (HM044084): Karanlık [15]; SX17 (FJ028608): Karanlık [2]; SX18 (FJ028630): Karanlık [3]; SX19 (HM044085): Obruk [1]; SX20 (HM044086): Obruk [1]; SX21 (HM044087): Zindan [2]; SX22 (HM044088): Karanlık [1]; SX23 (FJ028623): Zindan [1]; SX30 (EU332362); Çatdere [1]; SX32 (EU332365): Çatdere [2]; SX42 (HM044089): Obruk [1]; SX60 (HM044090): Zindan [1]; PX01 (HM044091): Armutludelik [5], Asarini [1], Delikli [2], Epçik [1]; PX02 (HM044092): Armutludelik [2], Asarini [1], Delikli [3], Epçik [1]; PX03 (HM044093): Armutludelik [2], Asarini [17], Delikli [12], Epçik [11]; PX04 (HM044094): Delikli [1]; PX05 (FJ028632): Epçik [1]; PX06 (FJ028642): Epçik [1]; PX07 (HM044095): Epçik [1]; PX08 (FJ028637): Epçik [1]; PX09 (HM044096): Armutludelik [1]; PX10 (HM044097): Armutludelik [11]; PX11 (HM044098): Armutludelik [2]; PX12 (HM044099): Armutludelik [7], Asarini [1], Epçik [1]; PX21 (HM044100): Armutludelik [1], Delikli [1]; PX22 (HM044101): Epçik [1]; PX23 (HM044102): Epçik [1]; PX24 (HM044103): Delikli [1].
